# Impaired socio-emotional processing in a developmental music disorder

**DOI:** 10.1038/srep34911

**Published:** 2016-10-11

**Authors:** César F. Lima, Olivia Brancatisano, Amy Fancourt, Daniel Müllensiefen, Sophie K. Scott, Jason D. Warren, Lauren Stewart

**Affiliations:** 1 Institute of Cognitive Neuroscience, University College London, London, UK; 2 Department of Psychology, Goldsmiths, University of London, London, UK; 3 Dementia Research Centre, Institute of Neurology, University College London, London, UK; 4 Center for Music in the Brain, Department of Clinical Medicine, Aarhus University & Royal Academy of Music, Aarhus/Aalborg, Denmark

## Abstract

Some individuals show a congenital deficit for music processing despite normal peripheral auditory processing, cognitive functioning, and music exposure. This condition, termed congenital amusia, is typically approached regarding its profile of musical and pitch difficulties. Here, we examine whether amusia also affects socio-emotional processing, probing auditory and visual domains. Thirteen adults with amusia and 11 controls completed two experiments. In Experiment 1, participants judged emotions in emotional speech prosody, nonverbal vocalizations (e.g., crying), and (silent) facial expressions. Target emotions were: amusement, anger, disgust, fear, pleasure, relief, and sadness. Compared to controls, amusics were impaired for all stimulus types, and the magnitude of their impairment was similar for auditory and visual emotions. In Experiment 2, participants listened to spontaneous and posed laughs, and either inferred the authenticity of the speaker’s state, or judged how much laughs were contagious. Amusics showed decreased sensitivity to laughter authenticity, but normal contagion responses. Across the experiments, mixed-effects models revealed that the acoustic features of vocal signals predicted socio-emotional evaluations in both groups, but the profile of predictive acoustic features was different in amusia. These findings suggest that a developmental music disorder can affect socio-emotional cognition in subtle ways, an impairment not restricted to auditory information.

Most humans have a propensity for music from early life. Newborns respond to changes in key and show hemispheric specialization for music^1^; and 5 to 24 month infants coordinate body movements to music, with the degree of coordination correlating with positive affect^2^. Deeply rooted in social interactions, exposure to music and engagement with music activities throughout childhood sets up sophisticated music processing systems, even in individuals without explicit training^3^,^4^,^5^. However, a minority of individuals fail to normally develop musical abilities despite normal peripheral auditory processing, intellectual functioning, and music exposure^6^. This neurodevelopmental condition is termed congenital amusia, and is characterized by lifelong impairments in music perception and production^7^. Those with congenital amusia have difficulty detecting out-of-tune notes in melodies, recognizing and memorizing melodies, singing in tune, discriminating changes in pitch direction, and perceiving melodic contours^6^,^7^,^8^,^9^. Amusia affects around 4% of the general population^10^ (but see^11^), and has an estimated heritability of 39%^12^. The majority of individuals with amusia are less interested in music than controls, employ music less in everyday situations, and report impoverished affective experiences in response to music^13^.

A central debate in amusia research concerns the implications of this disorder beyond the music domain. Individuals with congenital amusia do not usually report other difficulties, indicating that the most prominent manifestations are domain-specific. However, it has been suggested that the abnormal encoding of musical pitch in amusia reflects a generic pitch processing difficulty^8^,^14^. Subtle aspects of speech can be impaired, namely those requiring fine-grained pitch processing, including statement-question discrimination^8^,^15^, lexical tone perception^16^,^17^, phonological and phonemic awareness^18^, and prosody imitation^8^.

Relevant to the current study is the observation that amusia also affects the processing of emotional speech prosody. Thompson *et al*.^19^ compared 12 adults with amusia and 12 controls in a forced-choice emotion recognition task, using semantically neutral spoken utterances that conveyed emotions via prosodic cues only (happy, tender, afraid, irritated, sad, and neutrality). As a group, amusic individuals showed reduced accuracy for all categories apart from afraid and neutrality, and reported increased difficulties understanding others’ feelings from vocal cues in everyday life. Lolli *et al*.^20^ further showed that emotional prosody impairments in amusia might be more apparent when the amount of information in the speech signal is reduced using a low-pass filter. These findings support the idea of shared mechanisms between music and vocal emotions, and provide a mirror to the enhancements of emotional prosody recognition seen in musicians^21^.

The extent to which emotion processing impairments in amusia are limited to emotional speech prosody remains undetermined. Given the long line of research approaching amusia mostly in terms of musical and pitch deficits, it is parsimonious to expect emotion impairments to be restricted to auditory stimuli with a direct link to music or pitch processing, as in the case of prosody^22^. However, in everyday communication, socio-emotional stimuli are typically multimodal; the ability to recognize different auditory and visual socio-emotional cues is linked in normative development^23^,^24^ and involves modality-independent neural mechanisms to an important extent^25^. It is thus possible that, throughout development, emotion impairments initially related to pitch and music deficits lead to cascading effects that impact higher-order modality-independent components of socio-emotional cognition. Consistent with this, musicians show stronger responses to emotional speech prosody than non-musicians in regions implicated in modality-independent inferences about others’ mental states, including the medial prefrontal and anterior cingulate cortices^26^. Additionally, training studies indicate that musical activities enhance aspects of infants’ socio-emotional functioning beyond music/pitch processing, including the understanding of others’ actions based on vocal and visual affective cues^27^, and nonverbal communication and social interaction skills^28^.

Following on from these considerations, the present study addresses two novel questions. In Experiment 1, we ask if emotion recognition impairments in amusia are specific to emotional speech prosody, or generalize across different auditory and visual socio-emotional cues. In three tasks, thirteen adults with congenital amusia and 11 controls judged emotions in emotional speech prosody (using semantically neutral utterances), nonverbal vocalizations (e.g., crying), and (silent) facial expressions. Examining nonverbal vocalizations allows us to establish the generalizability of the link between amusia and vocal emotions. Emotions in nonverbal vocalizations and speech prosody both involve variations in acoustic cues such as pitch, but they can be distinguished regarding their underlying articulatory mechanisms^29^, and nonverbal vocalizations further reflect a primitive and universal form of communication^30^. Examining facial expressions establishes whether the emotion deficit is auditory-specific, or whether it reflects a failure at a modality-independent level of processing. In Experiment 2, we ask if the emotion impairment seen in amusia affects more nuanced socio-emotional inferences, beyond the recognition of emotion categories. The same participants completed two laughter perception tasks: they listened to spontaneous/genuine and posed laughs, and either evaluated their emotional authenticity, which requires inferring the state of the speaker; or judged their contagiousness, which requires a more automatic evaluation of subjective responses. It was recently shown that typical listeners are adept at judging the emotional authenticity of laughter, and neural responses to laughter in medial prefrontal cortex (associated with modality-independent inferences about mental states) predict this ability^31^. Across the two experiments, we examined how the two groups of participants used the acoustic features of the vocal signals during socio-emotional evaluations, in order to determine if putative emotion difficulties in amusia relate to atypical processing of acoustic information, namely pitch cues.

If the previously described deficits in emotional speech prosody recognition in amusia reflect general abnormalities in socio-emotional processing, at a modality-independent level of processing, we can predict: impaired performance, relative to controls, across different types of vocal stimuli and for facial expressions; and impaired interpretation of nuanced social information from laughter, particularly for inferences of others’ states (emotional authenticity).

## Results

### Experiment 1: Emotion recognition across modalities

Emotional speech prosody stimuli, nonverbal vocalizations and dynamic facial expressions were presented in sequential blocks as separate tasks. Seven emotion categories were investigated (amusement, anger, disgust, fear, pleasure, relief, and sadness), and participants completed a multidimensional rating procedure: they indicated how much each stimulus expressed the seven possible emotions on 7-point rating scales (for similar procedure^32^,^33^,^34^,^35^).

#### Recognition accuracy and ambivalent responses

**Figure 1:**
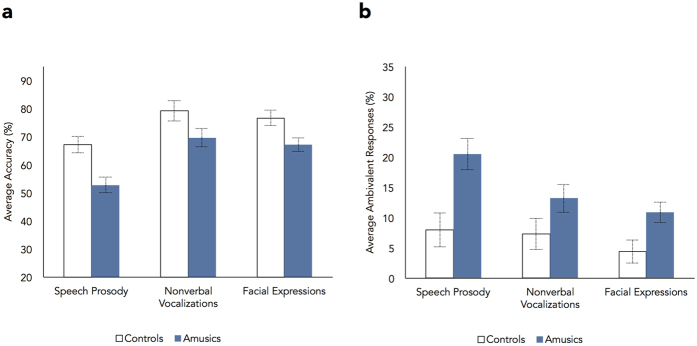
Percentage of correct responses (**a**) and ambivalent responses (**b**) as a function of group and emotion recognition task. Values are collapsed across emotion categories. Error bars indicate standard errors of the means. Amusics showed significantly reduced accuracy and provided more ambivalent responses than controls across the three tasks.

Performance was first analysed in terms of selectivity of responses, i.e., regarding how clearly participants distinguished between emotion categories. Two measures were extracted: (1) percentage of *correct* responses, reflecting cases where participants provided the highest rating on the scale corresponding to the intended emotion of the stimulus, and lower ratings on all the remaining scales; and (2) percentage of *ambivalent* responses, reflecting cases where participants rated two or more categories as the highest for a given stimulus, not being able to identify one single category as the most salient (e.g., 7 on the amusement and 7 on the relief scales)^33^,^35^. Correct and ambivalent responses are depicted in Fig. 1 for each group and task (complete confusion matrices are presented in Supplementary Information Tables S1–S3).

A mixed-design ANOVA was conducted on accuracy rates, including Group as between-subjects factor (controls, amusics), and Task (speech prosody, nonverbal vocalizations, faces) and Emotion (seven emotion categories) as repeated-measures factors. The main effect of group was significant (*F*[1,22] = 12.23, *p* = 0.002, 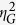 = 0.08, small effect), indicating that amusics had lower accuracy (*M* = 63.2%) than controls (*M* = 74.3%). The effects of emotion (*F*[6,132] = 11.08, *p* < 0.001, 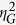 = 0.14, medium effect) and task (*F*[2,44] = 23.63, *p* < 0.001, 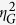 = 0.12, small effect) were also significant, but they did not interact with group (interaction Group × Task, *F*[2,44] = 0.09, *p* = .91, 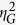 = 0.002; interaction Group × Emotion, *F*[6,132] = 0.98, *p* = 0.44, 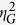 = 0.01; interaction Group × Emotion × Task, *F*[12,264] = 1.37, *p* = 0.18, 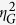 = 0.02). Group differences in accuracy were thus similar across emotions and tasks: amusics showed a similar impairment for vocal and facial stimuli.

In addition to showing reduced recognition accuracy, amusics gave more ambivalent responses (*M* = 14.9%) than controls (*M* = 6.6%) across tasks, as can be seen in Fig. 1b. This was supported by a main effect of group (*F*[1,22] = 11.85, *p* = 0.002, 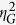 = 0.12, small effect). The effects of emotion (*F*[6,132] = 2.98, *p* = 0.009, 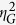 = 0.03, small effect) and task (*F*[2,44] = 7.65, *p* = 0.001, 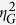 = 0.05, small effect) were significant, but they were independent of group (interaction Group × Task, *F*[2,44] = 1.26, *p* = 0.29, 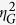 = 0.01; interaction Group × Emotion, *F*[6,132] = 0.63, *p* = 0.71, 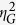 = 0.01; interaction Group × Emotion × Task, *F*[12,264] = 1.29, *p* = 0.22, 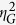 = 0.02). This provides further evidence for reduced selectivity of emotion responses in amusia.

Group differences in recognition accuracy and ambivalent responses cannot be explained by the numerical trend towards longer musical training in controls (Table 1), as they remained significant when musical training was included in the ANOVAs as a covariate (main effects of group, *p*s < 0.003; interactions between group and the other factors, *p*s > 0.14).

#### Emotion ratings across scales

**Figure 2:**
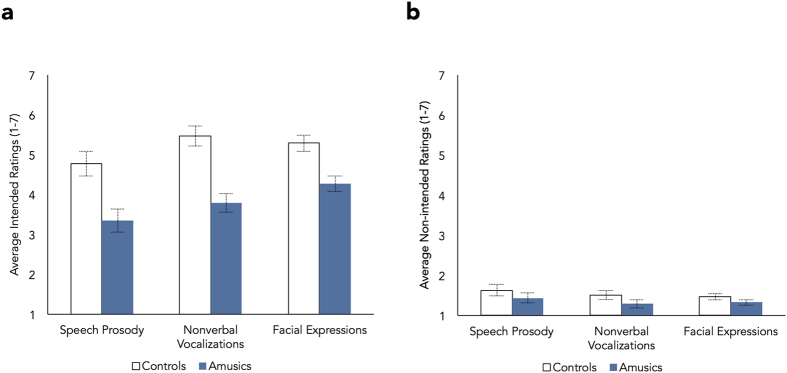
Average ratings provided on the intended ‘correct’ scales (**a**) and on the non-intended ‘incorrect’ scales (**b**) as a function of group and emotion recognition task. Values are collapsed across emotion categories. Error bars indicate standard errors of the means. Amusics showed significantly reduced sensitivity to the correct emotions, but not to the incorrect ones, across tasks.

Group differences in selectivity of responses, as defined in terms of lower accuracy and higher ambivalent responses in the amusic group, could result from two factors or their combination: lower ratings on the scale corresponding to the intended emotion (lower sensitivity); or higher ratings on the scales corresponding to the non-intended emotions. The average ratings on the intended and non-intended scales are presented in Fig. 2 (complete confusion matrices are presented in presented in Supplementary Information Tables S4–S6). An ANOVA on the intended ratings revealed that amusics (*M* = 3.8) showed lower sensitivity than controls (*M* = 5.2) to the intended emotions (main effect of group, *F*[1,22] = 19.82, *p* < 0.001, 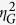 = 0.29, large effect). Effects of emotion (*F*[6,132] = 14.35, *p* < 0.001, 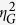 = 0.11, small effect) and task were found (*F*[1.5,32.95] = 12.3, *p* < 0.001, 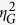 = 0.08, small effect), but none of them interacted with group (interaction Group x Task, *F*[1.5,32.95] = 2.36, *p* = 0.12, 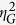 = 0.02; interaction Group x Emotion, *F*[6,132] = 1.21, *p* = 0.3, 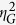 = 0.01; interaction Group × Emotion × Task, *F*[7.65,168.27] = 1.27, *p* = 0.26, 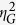 = 0.00).

By contrast, the magnitude and pattern of ratings across the non-intended scales was largely similar across groups, as indicated by 2 (Group) × 3 (Task) × 6 (Non-intended Ratings) ANOVAs conducted for each category. For amusement, anger, fear and pleasure, the main effect of group and the interactions between group and the other factors were non-significant (*p*s > 1). For disgust, non-intended ratings were similar across groups for all scales (*p*s > 0.11) apart from anger, on which controls provided higher ratings (*M* = 2.28) than amusics (*M* = 1.77, *p* = 0.05). For relief, controls provided higher ratings (*M *=* *2.8) than amusics (*M *=* *1.69, *p* = 0.03) on the pleasure scale for facial expressions, but groups were similar across the remaining conditions (*p*s > 0.1). For sadness, groups were similar (*p*s > 0.09), except that controls provided higher ratings (*M* = 2.4) than amusics (*M* = 1.77) on the fear (*p* = 0.03) and pleasure scales (controls, *M* = 1.39; amusics, *M* = 1.08, *p* = 0.007). This indicates that the reduced selectivity of emotion recognition in amusia is primarily a consequence of reduced sensitivity to the intended emotions.

#### Differences in vocal emotion recognition as a function of acoustic cues

Abnormalities in vocal emotion recognition in amusia may stem from difficulties tracking acoustic variations in the stimuli – participants with amusia may rely on acoustic information (particularly pitch^7^,^8^,^9^) to a lesser degree when judging vocal emotions. To examine this possibility, vocal stimuli were measured for pitch attributes, including fundamental frequency (F0) mean, standard deviation, minimum, maximum, and direction; they were also measured for non-pitch attributes, including duration, intensity, and spectral centre of gravity (for details, Materials and Methods). We then conducted logistic mixed-effects regression models, separately for controls and amusics, and examined whether the two groups relied similarly upon acoustic cues when providing their responses. For emotional speech prosody and nonverbal vocalizations, one mixed-effects model was conducted for each emotion: the acoustic attributes of the stimuli were entered as fixed effects predictor variables, and recognition accuracy (correct/incorrect) as dependent variable, while participants were used as a random effects variable. To keep the set of predictors small and to avoid collinearity, among the extracted acoustic features, we excluded those that were strongly intercorrelated (*r* > 0.6). F0 *M*, F0 *SD*, F0 direction, duration, intensity, and spectral centre of gravity were included, and F0 minimum and maximum were excluded. Models including all six acoustic features were specified first, after which we employed a backwards model selection procedure based on the second order Aikake Information Criterion^36^, which resulted in reduced models only featuring the acoustic attributes that contributed substantially to participants’ responses. The main findings are summarized in Table 2. Both for speech prosody and nonverbal vocalizations, the accuracy of the models was generally high in amusics (*M* = 0.73; range = 0.47–0.90) and controls (*M *=* 0*.80; range = 0.59–0.93), and they were significant for most emotions, indicating that responses could be reliably predicted from the acoustic cues. Prediction accuracy was slightly lower for amusics relative to controls, a finding consistent with the observed group differences in emotion recognition. However, this cannot be explained by an impaired use of individual acoustic cues in amusia. As can be seen in Table 2, each of the cues (pitch and non-pitch ones) predicted responses for at least three emotions in the amusia and control groups, revealing that participants in both groups used all the acoustic parameters. Importantly, an impaired ability to use a given cue would predict systematic group differences (in the same direction across emotions) for that cue, which we did not find. For instance, F0 mean predicted responses for pleasure in speech in controls but not in amusics, which would suggest a reduced use of this cue in amusia, but the same cue predicted responses to sadness in amusics but not in controls, indicating that amusia participants can use it as well as controls. Instead, our findings suggest group differences in how multiple acoustic cues are combined during emotion recognition, and in the qualitative pattern of the used cues: the number of acoustic predictors for each emotion was generally higher in controls (*M *=* *2.71) relative to amusic participants (*M *=* *1.86), and there were differences across groups in the specific combination of cues retained in each model for all emotions, both for speech prosody and for nonverbal vocalizations (see Table 2). These qualitative differences suggest an atypical mapping between acoustic features and the perceived emotional meaning of vocal expressions in amusia.

#### Associations between individual differences in diagnostic measures and in emotion recognition

We also examined how individual variation in the Montreal Battery for Evaluation of Amusia (MBEA^37^), a diagnostic measure, predicts accuracy on each emotion recognition task separately. A composite MBEA score was used, based on the three pitch-based MBEA subtests (scale, contour and interval scores; Table 1). A second set of analyses was conducted to examine links between performance on a pitch direction detection task (for details^8^,^38^), on which amusics showed impaired performance (Table 1), and emotion recognition accuracy.

MBEA scores positively predicted emotion recognition accuracy in speech prosody (*r*[22] = 0.58, *p* = 0.002), nonverbal vocalizations (*r*[22] = 0.43, *p* = 0.04), and facial expressions (*r*[22] = 0.43, *p* = 0.03; see Supplementary Information Figure S1a–c). Crucially, Cook’s distance values were below the critical value *F*[0.5,2,22] = 0.72 (see ref. 39), indicating that these associations cannot be explained by extreme data points on the regression models (Cook’s distance range = 0.00–0.34 for speech prosody, 0.00–0.27 for nonverbal vocalizations, and 0.00–0.47 for facial expressions). Lower thresholds on the pitch direction task (better performance) predicted higher emotion recognition accuracy in speech prosody (*r*[21] = −0.36, *p* = 0.09; Cook’s distance range = 0.00–1.24; after removing the data point with excessive Cook’s distance value, *r*[20] = −0.45, *p* = 0.03) and in facial expressions (*r*[21] = −0.53, *p* = 0.01; Cook’s distance range = 0.00–1.14; after removing the data point with excessive Cook’s distance value, *r*[20] = −0.57, *p* = 0.01; Supplementary Information Figure S1d–f). The association only approached significance for nonverbal vocalizations (*r*[21] = 0.39, *p* = 0.07; Cook’s distance range = 0.00–0.56). When these analyses were conducted on the smaller samples of controls and amusics separately, the associations were in the same direction as in the entire sample, but they did not reach significance (*p*s > 0.1).

### Experiment 2: Responses to spontaneous and posed laughter

Posed laughs, spontaneous laughs, and distractor emotional sounds were intermixed and presented to participants twice, for evaluations of authenticity and contagion. Participants used 7-point rating scales to indicate whether each vocalization reflected a posed or a genuinely felt emotion, and the extent to which it was contagious.

#### Evaluations of emotional authenticity and contagion

**Figure 3:**
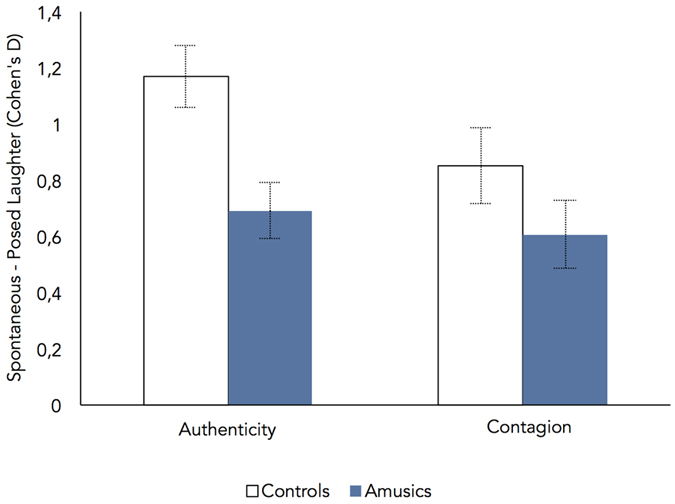
Magnitude of the difference between spontaneous and posed laughter as a function of group and task, i.e., difference between average authenticity and contagion ratings provided to spontaneous laughter, and average authenticity and contagion ratings provided to posed laughs (as expressed in terms of effect size, Cohen’s d). Error bars indicate standard errors of the means. Amusics showed significantly reduced sensitivity to laughter authenticity, but not to laughter contagiousness.

Performance was first analysed in terms of the magnitude of the perceived distinction between the two types of laughter. For each participant, and separately for authenticity and contagion, we computed a measure of effect size (Cohen’s d) indicating the standardized difference between average ratings provided to spontaneous laughs and average ratings provided to posed laughs; higher values indicate better discrimination between the two types of laughter. These effects are shown in Fig. 3. For authenticity, the magnitude of the distinction was smaller in the amusia than in the control group (*F*[1,18] = 10.295, *p* = 0.005, 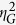 = 0.36, large effect). For contagion, the two groups were similar (*F*[1,20] = 1.81, *p* = 0.19, 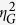 = 0.08).

Group differences in authenticity discrimination can result from differences in the perception of posed laughs, spontaneous laughs, or both. To approach this question, a 2 (Group) x 3 (Condition: posed laughter, spontaneous laughter, distractors) ANOVA was conducted. Average ratings across stimulus type are presented in Supplementary Information Table S7. There were differences between groups, but these varied across conditions (interaction Group × Condition, *F*[2,36] = 7.06, *p* = 0.003, 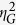 = 0.1, small effect; main effect of condition, *F*[2,36] = 95.54, *p* < 0.001, 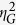 = 0.59, large effect; main effect of group, *F*[1,18] = 0.91, *p* = 0.35, 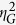 = 0.04). The two groups rated the authenticity of posed laughter (*F*[1,19] = 0.56, *p* = 0.46, 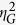 = 0.03) and distractors similarly (*F*[1,19] = 0.27, *p* = 0.61, 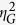 = 0.02), but for spontaneous laughter amusics provided lower ratings than controls (*F*[1,19] = 6.65, *p* = 0.02, 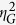 = 0.27, large effect). An ANOVA on contagion ratings revealed that groups were similar across conditions (main effect of group *F*[1,20] = 2.56, *p* = 0.13, 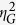 = 0.09; interaction Group × Condition, *F*[2,40] = 1.09, *p* = 0.35, 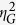 = 0.05). These analyses indicate that the perception of laughter authenticity – but not contagion – is altered in amusia, mostly due to reduced sensitivity to the authenticity of spontaneous laughs.

#### Differences in authenticity and contagion ratings as a function of acoustic cues

To investigate how pitch and non-pitch cues contributed to performance, linear mixed-effects models were conducted, taking F0 *M*, F0 *SD*, F0 direction, duration, intensity, and spectral centre of gravity as fixed effects, and authenticity and contagion ratings as dependent variables. As can be seen in Table 3, acoustic cues significantly predicted ratings across all conditions, apart from authenticity ratings of spontaneous laughs in amusia, a condition where group differences in sensitivity to authenticity were also more apparent. Consistent with the findings for the emotion recognition tasks, each of the cues predicted responses for at least one condition in both groups (apart from Intensity), and we found no evidence of systematic group differences across conditions for particular cues, indicating again a preserved ability to make use of individual acoustic parameters. Regarding contagion models, the number of acoustic cues that predicted ratings was similar across groups, and the specific combination of cues included in the models was also largely overlapping (Table 3). This is consistent with the absence of group differences in perceived contagion of laughter. Regarding authenticity models, the combination of cues included in the models was only partly overlapping across groups for posed laughs, and for spontaneous laughs none of the cues substantially contributed to ratings in the amusia group, as compared to two contributing cues in the control group.

#### Associations between individual differences in diagnostic measures and in laughter perception

MBEA scores positively predicted the ability to detect laughter authenticity (*r*[18] = 0.62, *p* = 0.003; Cook’s distance range = 0.00–0.35), but not laughter contagiousness (*r*[20] = 0.32, *p* = 0.15; Cook’s distance range = 0.00–0.09; Supplementary Information Figure S2a–b). The association between thresholds in the pitch direction task and laughter perception was not significant (authenticity, *r*(17) = −0.16, *p* = 0.51; Cook’s distance range = 0.00–0.62; contagiousness, *r*(19) = 0.02, *p* = 0.92; Cook’s distance range = 0.00–1.10). Separate analyses for controls and amusics also failed to yield significant results (*p*s > 0.1).

## Discussion

We examined how congenital amusia affects emotion processing in emotional speech prosody, nonverbal vocalizations, and facial expressions, considering the recognition of emotion categories and more nuanced social inferences from posed and spontaneous laughter. We present four novel findings. First, amusic participants showed reduced recognition of emotion categories in vocal expressions, not only in speech prosody, but also in nonverbal vocalizations. Second, emotion recognition abnormalities extended beyond the auditory domain, to (silent) dynamic facial expressions. Third, when processing laughter, amusics showed reduced sensitivity to emotional authenticity, but normal contagion responses. Fourth, mixed-effects models revealed that pitch and non-pitch features of the vocal signals predicted socio-emotional responses in both groups, but the constellation of acoustic predictors of performance was qualitatively different in amusia.

Several previous studies found that amusia can be associated with subtle impairments outside the music domain, but these have mostly focussed on speech processing^8^,^15^,^16^,^17^,^18^,^40^. How this disorder might impact the processing of different socio-emotional signals remains poorly understood. We corroborate the finding by Thompson *et al*.^19^ that amusia is associated with impaired interpretation of emotional speech prosody (see also ref. 20). While Thompson *et al*.^19^ observed that the effect may not generalize across emotions, namely for fear, we found no evidence of a group by emotion interaction. For fear, amusics scored 20% lower than controls. The discrepancy may relate to differences in the stimulus set or in the task; our task involved graded judgments across multiple dimensions of the stimuli, and this procedure may be more sensitive than the previously used forced-choice format. Crucially, we show for the first time that the amusic emotion impairment extends to purely nonverbal emotional vocalizations. These are similar to speech prosody in that they communicate emotions through temporal, amplitude, pitch, and spectral cues^41^, but they also show differences, namely regarding their production mechanisms and acoustic profiles – while prosody is constrained by linguistic information, nonverbal vocalizations are produced without language-related constrains. Our findings indicate that the link between amusia and vocal emotions is general, regardless of whether they are superimposed on language or not. An important consideration is whether these impairments truly reflect reduced emotion sensitivity or reduced confidence. It was argued before that reduced confidence is a possible origin of amusics’ difficulty in explicitly detecting out-of-key tones in melodies, as objective measures indicate that they can implicitly detect these changes^42^,^43^,^44^. However, this is an unlikely explanation for our findings. Reduced confidence would have arguably lead to group differences across all tasks and conditions, which we did not observe: on the emotion recognition tasks, amusics’ ratings of non-intended emotional expressions were largely similar to controls’, and on the laughter tasks the two groups performed similarly on ratings of contagion. Additionally, our measure of accuracy, which has been widely used in previous work^32^,^33^,^35^, is based on relative differences between ratings on the intended and non-intended scales (rather than on absolute values), and it therefore controls for possible general differences in how participants use the scales. The observation that amusic participants performed similarly to controls in several vocal processing conditions also indicates that their emotion impairments are unlikely to reflect a non-specific difficulty in processing dynamic (time-dependent) information, since such a general difficulty would arguably lead to impairments in all conditions, as they all involved dynamic stimuli.

Our findings suggest that the vocal emotional deficit in amusia cannot be reduced to an impaired ability to use individual pitch or non-pitch vocal cues during emotion judgments. In general, a range of pitch, amplitude, temporal and spectral vocal cues predicted responses for speech prosody and nonverbal vocalizations, a result consistent with previous studies^32^,^41^,^45^. Importantly, though, prediction accuracy was generally high across groups; additionally, each of the cues predicted responses for at least three emotions both in amusic and control participants, and amusic participants did not show a systematic reduced use of particular cues across emotions. Instead, our results point to a qualitatively different mapping between acoustic features and perceived emotions in amusia, at a higher-order integrative level of processing: compared to controls, amusic participants used a smaller number of cues during vocal emotion recognition, and the specific pattern of predictors was atypical. Such a dissociation between (relatively intact) implicit tracking of low-level acoustic information and (impaired) higher-order integrative processes can be related to previous findings. Hyde *et al*.^46^ found that the auditory cortex of amusics tracks pitch differences in melodies similarly to controls, the distinguishing feature being in functional connectivity between the auditory and inferior frontal cortices. Peretz *et al*.^42^ found that amusics’ can track quarter-tone pitch differences (as indicated by an early right-lateralized negative brain response), but fail to detect pitch anomalies in an explicit task (see also ref. 44).

Expanding current ideas on the effects of amusia outside the music domain, our study indicates that abnormalities in higher-order processing may extend to modality-independent components of socio-emotional cognition. Strong evidence for this argument comes from the observed impairment in processing facial emotional expressions, despite normal performance in a standardized test of perceptual abilities for faces (see Materials and Methods). We found significant group differences in recognition accuracy for dynamic facial expressions (10% on average), which were accompanied by additional differences in the proportion of ambivalent responses, indicating that amusic individuals have less differentiated emotion categories for visual stimuli. Note that the magnitude of group differences in accuracy was similar for faces and for auditory stimuli (10% vs. 14% for speech prosody and 10% for nonverbal vocalizations). They were also similar to the magnitude of prosodic impairments reported by Thompson *et al*.^19^, 10%. Adding to the robustness of the effect, individual differences on the MBEA and on a pitch direction detection task correlated with emotion recognition accuracy even when the three modalities were analysed separately. A similar correlation between pitch discrimination thresholds and emotion recognition accuracy was also recently reported by Lolli *et al*.^20^ for low-pass filtered speech prosody materials.

The results of the laughter perception tasks indicate that the emotion impairment in amusia is not restricted to the recognition of emotion categories. The ability of amusic participants to judge laughter authenticity was significantly reduced, and their use of acoustic features during authenticity evaluations was also atypical. Brain regions implicated in processing laughter include the medial prefrontal and cingulate cortices, superior temporal gyrus, pre-supplementary motor area, middle frontal gyrus, and putamen^31^. It is interesting to note that some of these regions, namely the cingulate cortex, superior temporal gyrus, middle frontal gyrus, and putamen, have been suggested to be abnormal in amusia^47^,^48^. Future studies will be needed to probe the links between neuroanatomical abnormalities in amusia and the impairments uncovered here. Regarding laughter contagion, no group differences were found. Amusics seem to evaluate their own emotional reactions similarly to controls, their impairments being most apparent when inferences about others are required.

We interpret our results as first evidence for a modality-independent impairment in socio-emotional processing in amusia. This does not question the ideas that the most prominent manifestations of this disorder are music-specific, and potentially related to a core fine-grained pitch processing deficit^6^,^7^,^8^,^9^,^47^. Our argument is that, throughout development, abnormalities in musical and pitch abilities may lead to subtle abnormalities in socio-emotional abilities that generalize beyond the auditory domain. The precise neural and developmental bases of this deficit need to be addressed in future studies. It remains to be determined whether the deficits reported here result from abnormalities in the same fronto-temporal pathways that have been argued to underlie music and pitch deficits in amusia^47^,^48^,^49^, or whether they relate to systems other than “music relevant” ones, that may also be abnormal in this disorder^48^. In addition to a role in music and pitch processing, fronto-temporal pathways are implicated in vocal and gestural communication^50^,^51^, social intelligence^52^, and the interpretation of mental affective states^53^. On the other hand, systems outside the music domain that might also be abnormal in amusia, including the anterior cingulate cortex, putamen, and medial orbital frontal gyri^48^, have been shown to support social cognition processes^54^,^55^ and higher-order aspects of vocal emotions^31^,^50^,^56^. Regarding developmental mechanisms, a recent hypothesis on the biological role of music suggests that pitch variations in vocalizations and their codification into music may have played a role in representing and conveying emotional mental states during human evolution and during infant development^57^,^58^. This hypothesis predicts a link between the development of music and the development of the ability to interpret emotional mental states in the human voice. Such a link is hypothesized to generalize beyond the voice, as the decoding of emotional states depends on modality-independent mechanisms to an important extent^23^,^25^. Of course our correlational design cannot establish a causal link between amusia and socio-emotional sensitivity. However, amusic individuals present themselves as having lifelong difficulties that are selective to music, and all the amusic participants in the current study perform substantially below the average of the general population (at least 2 *SD*) in objective tests of music perception. The socio-emotional impairments uncovered here are correlated with music capacities, and detectable in group-level comparisons, but they seem less prominent than the music difficulties. It is thus unlikely that they would be a cause, rather than a consequence, of amusia.

In the current work we were interested in the explicit processing of different types of social-emotional auditory and visual information, but in the future it will also be of interest to ask how this information is processed at an implicit level, and how auditory and visual emotional cues are combined when presented simultaneously. In a recent study, Lu *et al*.^44^ showed that individuals with amusia are biased to rely on unattended visual information (spatial position of dots) during auditory judgments (direction of pitch changes), but it remains unanswered if this will also be the case during socio-emotional judgements, considering our finding that visual emotional processing is impaired in amusia. Future studies will also shed light on how the deficits reported here might relate to the recognition of musical emotions in amusia. Which aspects of musical emotions are impaired in amusia, and which ones are preserved, remains poorly understood. Gosselin *et al*.^59^ reported only subtle deficits in the processing of musical emotions in a sample of participants with amusia, for the recognition of four basic emotion categories, happiness, sadness, fear and peacefulness. Given the heterogeneity in the amusic population, our results will need to be extended to different samples.

To conclude, the present study provides the first demonstration of a modality-independent deficit in socio-emotional processing in congenital amusia. Building on prior evidence, we established that amusics show impaired recognition of emotions in different types of vocal expressions. Crucially, we showed that the amusic emotion deficit extends to dynamic facial expressions and to inferences of emotional authenticity in laughter. Our findings suggest a novel link between music and higher-order components of socio-emotional cognition, and potentially have implications for our understanding of the socio-biological role of music.

## Materials and Methods

### Participants

Twenty-four participants were tested, including 13 amusics and 11 controls. They were all native speakers of British English, and none reported history of neurological, psychiatric or hearing disorders. Table 1 presents their demographic and background information. Amusics and controls were matched for age, sex, musical training, and education. They performed similarly in the National Adult Reading Test^60^ and in the digit span test of the Wechsler Adult Intelligence Scale-III^61^. The Cambridge Face Perception Test^62^ confirmed that amusics did not have impaired perceptual abilities for faces. Written informed consent was collected from all participants and ethical approval was obtained from the Departmental Ethics Committee, Department of Psychology, Goldsmiths. The experiments were performed in accordance with the relevant guidelines and regulations.

Amusia was diagnosed using the MBEA^37^. After completing an online session consisting of the scale and rhythm subtests, participants completed further on-site testing. They retook the scale and rhythm subtests, and were additionally administered the contour and interval subtests. A composite score was computed for the three pitch-based MBEA subtests, corresponding to the sum of the scale, contour and interval scores; participants performing more than 2 *SD* below the average for the normal population were confirmed as amusics (i.e., at or below 65/90^8^,^19^,^37^). The pitch-based subtests were emphasized for diagnosis because amusics’ performance on the rhythm subtest may be preserved^19^,^37^. We additionally determined participants’ thresholds for the detection of pitch changes and discrimination of pitch direction in pure tones (for details^8^,^38^). Amusics had higher thresholds than controls for pitch direction, but were normal for pitch change (Table 1).

### Emotion recognition tasks

#### Stimuli

The stimulus sets consisted of 105 spoken utterances varying in prosody, 105 purely nonverbal vocalizations, and 105 dynamic facial expressions, recorded and validated to express specific emotion categories. Seven emotions were investigated, 15 stimuli per emotion: amusement, anger, disgust, fear, pleasure, relief, and sadness.

The emotional speech prosody stimuli were selected from a newly recorded corpus (recording and validation procedures are presented in Supplementary Information). They consisted of semantically neutral spoken utterances that communicated emotions via variations in prosodic cues only. The set of 105 stimuli used here was selected based on validation data (mean recognition accuracy = 69.47%; *SD* = 11.66). The best possible match for emotion recognition accuracy across emotions was ensured: accuracy was lowest for sadness (60.67%) and highest for amusement (74.67%).

Nonverbal emotional vocalizations were selected from a validated corpus^32^. They consisted of brief vocal sounds without verbal content, such as screams, sobs, or sighs, as produced by two male and two female adults (mean recognition accuracy from validation data = 73.1%; *SD* = 18.38; accuracy was lowest for pleasure, 64.67%, and highest for disgust, 84.67%).

Facial expressions were selected from the Geneva Multimodal Emotion Portrayals Core Set corpus, that features 10 actors^63^. The actors were filmed and audio-recorded while producing expressions that involved facial, vocal and body cues simultaneously. We focussed on facial expressions only; the videos were muted and the body postures were not visible. In the validation procedure, the mean recognition accuracy for the selected 105 expressions was 70.29% (*SD* = 12.52; accuracy was lowest for pleasure, 60.67%, and highest for amusement, 80.67%). Their duration was 2,462 ms on average (*SD* = 1,304).

#### Procedure

The three sets of stimuli were presented in sequential blocks as separate tasks, and they were completed in a sound attenuated booth. The procedure was similar across tasks: the 105 stimuli were presented in a randomized order after a familiarization phase; on each trial, participants heard or saw a stimulus and provided ratings on seven 7-point scales, from 1 (*not at all*) to 7 (*very much*), indicating how much the emotions were expressed. Thus, a multidimensional procedure was used, i.e., the stimuli were evaluated regarding all possible emotions^32^,^33^,^34^,^35^. The list of emotions was presented on the computer screen after each stimulus, along with the rating scales; responses were collected via mouse clicks. The order of the emotions in the list was randomized across participants. Participants could decide the order of their ratings across the scales; however, if they noticed that one emotion was more prominently expressed than the others, they were encouraged to provide that rating first. E-Prime software (Psychology Software Tools, Inc., Pittsburgh, PA) controlled stimulus presentation and data collection. The auditory stimuli were presented via high quality headphones.

#### Acoustic Measurements

Speech prosody stimuli and nonverbal vocalizations were acoustically analysed using Praat software, version 5.4.05^64^. Eight parameters were extracted, covering pitch, timing, intensity and spectral domains: fundamental frequency (F0), including mean (Hz), standard deviation, minimum, maximum, and direction (rising/falling trends); duration (ms); intensity (dB); and spectral centre of gravity (Hz). Pitch direction was measured by the slope of the regression line across each stimulus, with a positive slope indicating a rising trend in F0 as a function of time, and a negative slope indicating a falling trend. Regarding intensity, variation across the whole waveform is reported (*SD*); mean values are not reported because the stimuli were normalized for intensity (root-mean-square amplitude) to control for the dynamic range of the raw recordings. These acoustic parameters are depicted in Table 4. There was wide variability in the stimulus sets for a range of cues, as expected according to previous studies^32^,^41^,^45^. These acoustic parameters provided sufficient information to predict the category of the stimuli: for speech prosody, a standard discriminant analysis including the acoustic parameters as independent variables and the category as dependent variable correctly categorized 55.2% of the stimuli (chance-level, 14.3%; Wilks’s *λ* = 0.23; *F*[48,451] = 3.22, *p* < 0.001); for nonverbal vocalizations, a similar model correctly categorized 60% of the stimuli (Wilks’s *λ* = 0.14; *F*[48,451] = 4.65, *p* < 0.001).

### Authenticity and contagion evaluations of laughter

#### Stimuli

The laughter stimuli consisted of 24 posed and 24 spontaneous laughs. They were generated by seven adults (four female) in a sound-proof anechoic chamber at University College London. Spontaneous laughter was elicited using an amusement induction situation: each speaker was shown video clips, which they identified beforehand as amusing and that would easily cause them to laugh aloud^31^,^65^. For posed laughter, the speakers simulated laughter in the absence of external stimulation, while trying to make the expression sound credible. The laughs were selected based on a validation study (*N* = 40; mean age = 23.6; *SD* = 4.8; none of these participants took part in the main study). On a seven-point rating scale (1–7), spontaneous laughs were perceived as more authentic (*M *=* *4.85; *SD* = 0.82) than posed laughs (*M *=* *3.43; *SD* = 0.82; *t*[46] = 5.7, *p* < 0.001). The laughs were intermixed with 18 distractors consisting of acted vocalizations expressing other emotions, including sadness, pleasure, relief and achievement^32^. These were included so that participants would be less likely to detect that the manipulation concerned laughter only; and so that we could examine whether group effects are selective to our manipulation or rather an unspecific effect in how participants rate the affective properties of sounds.

#### Procedure

The 48 laughs and 18 distractors were randomized and presented twice to each participant, as separate tasks, for authenticity and contagion evaluations. The order of the tasks was counter-balanced. For authenticity, participants rated how much the vocalizations reflected a genuinely felt emotion on a 7-point scale, from 1 (*the person is acting out the expression*) to 7 (*the person is genuinely feeling the emotion*). For contagion, participants rated how much the vocalizations were contagious, from 1 (*it does not make me feel like mimicking or feeling the emotion*) to 7 (*it makes me feel like mimicking or feeling the emotion*). MATLAB (Mathworks, Inc., Natick, MA), using the Psychophysics Toolbox Extension (http://psychtoolbox.org/) controlled stimulus presentation. Because of time restrictions, one control participant did not complete the two tasks, and three participants completed only one of them (for authenticity, there were missing data from 2 amusics and 1 control; for contagion, there was missing data from 1 amusic). The analyses of these tasks were conducted on reduced sample sizes.

#### Acoustic measurements

The laughs were measured concerning the same acoustic parameters as the stimuli used in the emotion recognition tasks (Table 4). Posed and spontaneous laughs were matched for duration (*t*[46] = −0.76, *p* = 0.45), and they differed regarding mean F0 (*t*[46] = −6.98, *p* < 0.001), minimum F0 (*t*[46] = −4.82, *p* < 0.001), maximum F0 (*t*[46] = −4.3, *p* < 0.001), and direction of pitch trajectory (*t*[46] = −2.43, *p* = 0.02). A standard discriminant analysis revealed that the acoustic parameters provide enough information to correctly categorize 93.8% of the laughs (chance-level, 50%; Wilks’s *λ* = 0.4; *F*[8,39] = 7.46, *p* < 0.001).

### Statistical analysis

Group differences were examined using mixed-design and one-way ANOVAs. Greenhouse-Geisser corrections were applied when necessary (Mauchly’s sphericity test). Analyses based on proportions were conducted after arcsine-root transformation. Effect sizes were calculated using generalized eta squared, 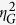 (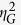
above 0.02 reflects a small effect, 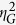 above 0.13 reflects a medium effect, and 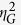
above 0.26 reflects a large effect^66^,^67^). Linear regression analyses were used to relate performance on emotion recognition with performance on the MBEA and with pitch direction thresholds. We calculated Cook’s distance values to inspect the possibility that some extreme subjects are driving the observed effects^39^. Exact *p* values are reported unless they are <0.001.

## Additional Information

**How to cite this article**: Lima, C.F. *et al*. Impaired socio-emotional processing in a developmental music disorder. *Sci. Rep.* **6**, 34911; doi: 10.1038/srep34911 (2016).

## Supplementary Material

Supplementary Information

## Figures and Tables

**Table 1:** Demographic and background characteristics of participants.

**Characteristics**	**Amusics (n = 13)**	**Controls (n = 11)**	***t***	***p***
Age (years)	57.92 (11.35)	53.18 (13.59)	−0.93	0.36
Sex	9F/4M	8F/3M	—	1
Handedness	13R/0L	9R/2L	—	0.20
Musical training (years)	0.85 (1.34)	2.30 (4.57)	0.11	0.29
Education (years)	15.92 (2.84)	15.64 (2.50)	−0.26	0.80
NART (words correctly read, /50)	44.00 (4.42)	44.40 (2.76)	0.24	0.81
Digit Span (raw scores)	21.00 (3.16)	20.09 (4.35)	−0.54	0.59
MBEA (correct responses)				
Scale (/30)	19.23 (2.71)	27.18 (2.36)	7.59	<0.001
Contour (/30)	19.77 (3.39)	27.73 (2.33)	6.57	<0.001
Interval (/30)	18.00 (2.00)	27.45 (2.38)	10.58	<0.001
Rhythm (/30)	24.46 (3.80)	28.45 (1.44)	3.28	0.003
Pitch Composite (/90)	57.00 (6.70)	82.36 (6.10)	9.62	<0.001
Pitch Change Detection Threshold (semitones)	0.31 (0.32)	0.16 (0.06)	−1.56	0.13
Pitch Direction Discrimination Threshold (semitones)	1.28 (1.46)	0.18 (0.08)	−2.50	0.02
CFPT (sum of errors)				
Upright (/94)	45.00 (14.55)	44.40 (7.82)	−0.12	0.91
Inverted (/94)	77.40 (15.06)	72.80 (13.17)	−0.73	0.48

*Note*. F = female; M = male; R = right; L = left; NART = National Adult Reading Test; MBEA = Montreal Battery of Evaluation of Amusia; CFPT = Cambridge Face Perception Test. Standard deviations are provided in parentheses. *t* values correspond to the statistic of independent samples t-tests (two-tailed, *df* = 22). For sex and handedness, groups were compared using Fisher’s exact test. There were missing data for some of the background measures, and for these mean, *SD* and *t-*tests were computed on reduced sample sizes: for the NART and CFPT, data were missing from three amusics and one control participants; for digit span, data were missing from three amusics; and for the pitch thresholds tasks, data were missing from one amusic.

**Table 2:** Mixed-effects regression models on the predictive value of acoustic cues for vocal emotion recognition.

**Task**	**Group/Emotion**	**Acoustic Predictors**						**Model accuracy**
		**F0**_**M**_	**F0**_**SD**_	**F0**_**direction**_	**Duration**	**Intensity**	**Spectral**_**COG**_	
Speech Prosody	Amusics							
	Amusement	−0.31	—	—	—	—	—	0.75
	Anger	—	—	—	—	0.52	0.67	0.65^*^
	Disgust	—	—	—	0.51	—	—	0.47^*^
	Fear	−0.39	—	—	—	—	0.96	0.63^*^
	Pleasure	—	—	0.46	—	—	—	0.80^*^
	Relief	−0.72	0.55	—	—	—	—	0.66^*^
	Sadness	1.19	−0.57	—	—	—	—	0.63^*^
	Controls							
	Amusement	−0.90	1.35	1.14	—	—	−2.88	0.76^*^
	Anger	—	0.40	—	—	0.46	—	0.81^*^
	Disgust	—	—	—	—	−0.39	—	0.59
	Fear	—	−0.79	—	2.10	−0.92	−0.82	0.76^*^
	Pleasure	−1.08	0.51	—	—	—	—	0.81^*^
	Relief	—	—	—	—	—	—	0.79
	Sadness	—	—	—	−0.69	1.29	0.72	0.77^*^
Nonverbal Vocalizations	Amusics							
	Amusement	0.71	—	0.95	—	—	1.23	0.83^*^
	Anger	—	—	−0.56	—	—	—	0.77^*^
	Disgust	—	—	—	—	—	—	0.90
	Fear	—	−0.51	—	—	0.34	—	0.78^*^
	Pleasure	—	—	−0.78	1.10	—	—	0.84^*^
	Relief	—	—	−0.34	0.61	−0.41	—	0.80^*^
	Sadness	−2.24	1.56	−0.63	—	—	2.77	0.77^*^
	Controls							
	Amusement	—	—	—	—	—	0.74	0.86^*^
	Anger	—	−0.84	−2.79	—	2.52	—	0.88^*^
	Disgust	−2.17	1.65	−1.02	—	0.66	—	0.93^*^
	Fear	1.77	—	—	0.62	1.56	−3.67	0.85^*^
	Pleasure	5.87	−2.27	—	3.70	—	−15.29	0.89^*^
	Relief	−1.07	—	−0.45	0.73	—	0.88	0.81^*^
	Sadness	−1.17	1.42	—	—	—	—	0.75^*^

*Note.* Values represent standardized regression coefficients for the acoustic cues retained in the model after the model selection procedure (empty cells indicate that the acoustic cue was not retained in the model). Model accuracy values represent the proportion of participant responses correctly classified by the model, including fixed and random effects. Each model was fitted to the full sample of amusic or control participants, across all stimuli for a given task and emotion; the final models contained between 0 (intercept and random effect only) and six fixed effects predictor variables. F0 = fundamental frequency; _COG_ = centre of gravity. ^*^p < 0.05, likelihood ratio test for the significance of the model with fixed effects (acoustic parameters) compared to a random-effects model only.

**Table 3:** Mixed-effects regression models on the predictive value of acoustic cues for authenticity and contagion evaluations of laughter.

**Task**	**Group/Emotion**	**Acoustic Predictors**						***R***^**2**^
		**F0**_**M**_	**F0**_**SD**_	**F0**_**direction**_	**Duration**	**Intensity**	**Spectral**_**COG**_	
Authenticity	Amusics							
	Posed	−0.64	0.71	−0.29	0.26	—	—	0.23^*^
	Spontaneous	—	—	—	—	—	—	0.14
	Controls							
	Posed	—	0.54	−0.29	—	—	—	0.18^*^
	Spontaneous	0.44	—	—	—	—	0.27	0.15^*^
Contagion	Amusics							
	Posed	−0.67	0.77	−0.42	0.28	—	—	0.49^*^
	Spontaneous	0.24	—	—	—	—	0.34	0.56^*^
	Controls							
	Posed	−0.35	0.54	−0.47	0.24	—	—	0.28^*^
	Spontaneous	0.51	−0.22	—	—	—	—	0.19^*^

*Note.* Values represent standardized regression coefficients for the acoustic cues retained in the model after the model selection procedure (empty cells indicate that the acoustic cue was not retained in the model). *R*^2^ values are conditional *R*^2^ values representing the amount of variance explained by the model, including fixed and random effects^68^,^69^. Each model was fitted to the full sample of amusic or control participants, across all stimuli for a given task and stimulus type; the final models contained between 0 (intercept and random effect only) and six fixed effects predictor variables. F0 = fundamental frequency; _COG_ = centre of gravity. *p < 0.05, likelihood ratio test for the significance of the model with fixed effects (acoustic parameters) compared to a random-effects model only.

**Table 4:** Acoustic characteristics of vocal emotional stimuli.

**Stimulus type/ Emotion**	**Acoustic cue**							
	**F0**_**M**_**(Hz)**	**F0**_**SD**_**(Hz)**	**F0**_**Min**_**(Hz)**	**F0**_**Max**_**(Hz)**	**F0**_**direction**_	**Duration (ms)**	**Intensity**_**SD**_**(dB)**	**Spectral**_**COG**_**(Hz)**
Speech Prosody								
Amusement	286.9	95.3	152.5	540.4	−0.33	2142	18.8	813
Anger	304.5	120.2	157.6	648	−0.15	2127	21.1	1104.4
Disgust	262.5	141.2	122.6	700.7	−0.11	3065	19.7	911
Fear	328	83.5	194	586.7	−0.17	1835	23.4	885.3
Pleasure	221.5	117.2	142.5	632.8	−0.12	2759	19.2	1051.5
Relief	246.2	105.6	133.3	543	−0.37	1965	22.3	786.8
Sadness	269	134.2	122.2	594.8	−0.2	2482	21.6	843.3
Nonverbal Vocalizations								
Amusement	364	143.8	185.6	661.1	−0.02	1068	10.8	881.4
Anger	222.5	96.9	104.8	473.6	−0.21	900	8.5	1039
Disgust	331.6	167.4	141.4	658.6	0.26	878	8.1	931.5
Fear	420	63.1	322.2	537.6	−0.28	877	13	948.6
Pleasure	199.6	90.3	123.7	479.6	−0.42	1029	7	251.1
Relief	450.7	119.8	280.6	623.2	0.3	872	9.6	1026.2
Sadness	278.3	103.9	169.4	559.7	−0.4	982	8.8	481.9
Laughter								
Posed	276	105.5	139.5	554.5	−0.29	2366	14.6	783.1
Spontaneous	467.9	132.4	249.4	780	−0.02	2439	14.8	944.1

*Note.* F0 = fundamental frequency; Min = minimum; Max = maximum; F0_direction_ = standardized regression coefficients reflecting changes in F0 over time; _COG_ = centre of gravity.
