# Supplementary material for: Impaired socio-emotional processing in a developmental music disorder
Source: Sci Rep. 2016 Oct 11;6:34911. doi: 10.1038/srep34911 (PMC5057155; doi:10.1038/srep34911)
Supplement: Supplementary Information [file srep34911-s1.doc]

**Supplementary Information**

Impaired socio-emotional processing in a developmental music disorder

César F. Lima^1*^, Olivia Brancatisano^2*^, Amy Fancourt^2^, Daniel Müllensiefen^2^,

Sophie K. Scott^1^, Jason D. Warren^3^, and Lauren Stewart^2,4^

^1^Institute of Cognitive Neuroscience, University College London, London, UK

^2^Department of Psychology, Goldsmiths, University of London, London, UK

^3^Dementia Research Centre, Institute of Neurology, University College London, London, UK

^4^Center for Music in the Brain, Department of Clinical Medicine, Aarhus University & Royal Academy of Music, Aarhus/Aalborg, Denmark

^*^ César F. Lima and Olivia Brancatisano have contributed equally to this work

Address correspondence to Dr. César F. Lima, Institute of Cognitive Neuroscience, University College London, 17 Queen Square, London WC1N 3AR, UK. E-mail: c.lima@ucl.ac.uk or cesarflima@gmail.com; or to Prof. Lauren Stewart, Department of Psychology, Goldsmiths, University of London, New Cross Road, New Cross, London SE14 6NW, UK. E-mail: l.stewart@gold.ac.uk

Table S1. Confusion matrices presenting correct categorizations, distribution of errors, and ambivalent responses for speech prosody. Values represent percentages.

|  | Distribution of responses (percentage) | | | | | | | |
| --- | --- | --- | --- | --- | --- | --- | --- | --- |
| Group/Emotion | Amusement | Anger | Disgust | Fear | Pleasure | Relief | Sadness | Ambivalent |
| Controls |  |  |  |  |  |  |  |  |
| Amusement | **72.9** | 2.6 | 0 | 0 | 0.7 | 14.2 | 3.2 | 6.5 |
| Anger | 2.6 | **71.6** | 7.4 | 1.3 | 1.6 | 7 | 0 | 8.5 |
| Disgust | 2.7 | 18.4 | **53.9** | 4.4 | 2.6 | 5.7 | 3.9 | 8.4 |
| Fear | 2.6 | 10.3 | 2.1 | **67.8** | 0 | 3.2 | 6.4 | 7.5 |
| Pleasure | 4.5 | 0 | 1.4 | 1.4 | **68** | 4.2 | 13.6 | 6.9 |
| Relief | 3.1 | 18 | 2.1 | 0 | 0.6 | **69.2** | 0.6 | 6.3 |
| Sadness | 1.9 | 0 | 0 | 14.5 | 1.3 | 3.5 | **67.1** | 11.7 |
| Amusics |  |  |  |  |  |  |  |  |
| Amusement | **64.6** | 2.6 | 2.3 | 2.3 | 1 | 7.8 | 3.2 | 16.1 |
| Anger | 3.3 | **48.2** | 10.3 | 5.4 | 3.9 | 9 | 0 | 19.9 |
| Disgust | 3.4 | 18.9 | **33.6** | 2.3 | 3.5 | 4.6 | 8.7 | 25.1 |
| Fear | 1.1 | 6.9 | 3.1 | **48.1** | 2.1 | 9.3 | 8.9 | 20.7 |
| Pleasure | 4.8 | 0 | 1 | 0 | **69.8** | 4.6 | 5.9 | 14 |
| Relief | 7.6 | 10 | 4.8 | 1.7 | 1.7 | **50.4** | 1.1 | 22.1 |
| Sadness | 2.2 | 0 | 1.4 | 8.6 | 3.7 | 3.5 | **55.2** | 25.4 |

Table S2. Confusion matrices presenting correct categorizations, distribution of errors, and ambivalent responses for nonverbal vocalizations. Values represent percentages.

|  | Distribution of responses (percentage) | | | | | | | |
| --- | --- | --- | --- | --- | --- | --- | --- | --- |
| Group/Emotion | Amusement | Anger | Disgust | Fear | Pleasure | Relief | Sadness | Ambivalent |
| Controls |  |  |  |  |  |  |  |  |
| Amusement | **80.3** | 0.6 | 0 | 2.5 | 1.5 | 1.9 | 7.3 | 6 |
| Anger | 0 | **82.5** | 7.9 | 0.6 | 0.6 | 1.5 | 0 | 7 |
| Disgust | 0.6 | 1.9 | **87.8** | 0 | 0.6 | 1.5 | 0 | 7.6 |
| Fear | 0 | 1.8 | 5 | **77** | 4.5 | 2.4 | 0.6 | 8.6 |
| Pleasure | 1.2 | 0 | 0 | 2 | **88.7** | 3.1 | 1.3 | 4.3 |
| Relief | 0 | 2.7 | 4.4 | 6.4 | 5.8 | **68.7** | 1.2 | 10.8 |
| Sadness | 0 | 0 | 2.5 | 12.5 | 5.3 | 3.1 | **69** | 7.6 |
| Amusics |  |  |  |  |  |  |  |  |
| Amusement | **75.7** | 0.5 | 0.5 | 1 | 0.5 | 2.4 | 5.2 | 14.1 |
| Anger | 0 | **68.9** | 10.7 | 2.1 | 1 | 5.1 | 0 | 12.2 |
| Disgust | 2.1 | 2.1 | **71.1** | 1.1 | 0.6 | 5.9 | 0 | 17.1 |
| Fear | 1.7 | 0 | 3.2 | **66.4** | 7.7 | 4.2 | 1 | 15.6 |
| Pleasure | 2.7 | 0.5 | 1.1 | 1.5 | **71.2** | 8.5 | 1.1 | 13.3 |
| Relief | 0.5 | 3.2 | 3.2 | 6.5 | 5.4 | **71.8** | 1.6 | 7.8 |
| Sadness | 2.1 | 1.1 | 1.1 | 15.2 | 1.6 | 3.7 | **63** | 12.2 |

Table S3. Confusion matrices presenting correct categorizations, distribution of errors, and ambivalent responses for facial expressions. Values represent percentages.

|  | Distribution of responses (percentage) | | | | | | | |
| --- | --- | --- | --- | --- | --- | --- | --- | --- |
| Group/Emotion | Amusement | Anger | Disgust | Fear | Pleasure | Relief | Sadness | Ambivalent |
| Controls |  |  |  |  |  |  |  |  |
| Amusement | **97.1** | 0 | 0 | 0 | 0 | 0.6 | 0 | 2.3 |
| Anger | 0 | **95.6** | 1.2 | 0.6 | 0 | 0 | 0 | 2.5 |
| Disgust | 0.6 | 3.1 | **66.8** | 5.2 | 0 | 2.8 | 12.7 | 8.7 |
| Fear | 0 | 4.5 | 0 | **94.2** | 0 | 0 | 0 | 1.3 |
| Pleasure | 38.7 | 0 | 0 | 0 | **39.8** | 15.5 | 1.3 | 4.8 |
| Relief | 0.6 | 1.3 | 2 | 0 | 20.6 | **71.6** | 2.7 | 1.3 |
| Sadness | 0.6 | 1.2 | 5.1 | 7.8 | 1.2 | 2.4 | **71.5** | 10.2 |
| Amusics |  |  |  |  |  |  |  |  |
| Amusement | **88.2** | 0.6 | 0 | 1 | 1.1 | 2.2 | 0.5 | 6.3 |
| Anger | 0.5 | **93.5** | 0 | 1.7 | 0 | 0 | 0 | 4.3 |
| Disgust | 1.6 | 5.5 | **50.9** | 3.2 | 0 | 0 | 26.6 | 12.3 |
| Fear | 0 | 9.7 | 1.2 | **75.6** | 0 | 0.5 | 1 | 11.9 |
| Pleasure | 40.9 | 0 | 0 | 0 | **31.4** | 12.1 | 1.1 | 14.5 |
| Relief | 3.6 | 1 | 2.7 | 0.5 | 10.7 | **58.9** | 7.6 | 14.9 |
| Sadness | 1.1 | 1.1 | 4.7 | 4.9 | 0 | 2.2 | **71.6** | 14.3 |

**Table S4. Average ratings across emotion scales as a function of emotion category for speech prosody. Values vary between 1 and 7.**

|  | Rating scale | | | | | | | Derived Accuracy |
| --- | --- | --- | --- | --- | --- | --- | --- | --- |
| Group/Emotion | Amusement | Anger | Disgust | Fear | Pleasure | Relief | Sadness |
| Controls |  |  |  |  |  |  |  |  |
| Amusement | **5** | 1.4 | 1.6 | 1.3 | 1.3 | 2.8 | 1.4 | 72.9 |
| Anger | 1.5 | **5** | 2.4 | 1.7 | 1.1 | 1.7 | 1.3 | 71.6 |
| Disgust | 1.5 | 3 | **4.4** | 1.6 | 1.1 | 1.6 | 1.6 | 53.9 |
| Fear | 1.5 | 2 | 1.7 | **4.7** | 1 | 1.6 | 2 | 67.8 |
| Pleasure | 1.7 | 1.1 | 1.4 | 1.3 | **4.7** | 2 | 2.2 | 68 |
| Relief | 2 | 2.2 | 1.6 | 1.2 | 1.3 | **4.7** | 1.3 | 69.2 |
| Sadness | 1.3 | 1.3 | 1.4 | 2.7 | 1.3 | 1.9 | **4.9** | 67.1 |
| Amusics |  |  |  |  |  |  |  |  |
| Amusement | **3.6** | 1.3 | 1.3 | 1.2 | 1.3 | 1.9 | 1.3 | 64.6 |
| Anger | 1.3 | **3.1** | 2.1 | 1.4 | 1.2 | 1.6 | 1.3 | 48.2 |
| Disgust | 1.4 | 2.3 | **3** | 1.4 | 1.2 | 1.3 | 1.6 | 33.6 |
| Fear | 1.2 | 1.7 | 1.5 | **3.2** | 1.1 | 1.5 | 2 | 48.1 |
| Pleasure | 1.6 | 1.1 | 1.2 | 1.1 | **3.7** | 1.7 | 1.4 | 69.8 |
| Relief | 1.7 | 1.6 | 1.4 | 1.2 | 1.3 | **3.2** | 1.3 | 50.4 |
| Sadness | 1.2 | 1.3 | 1.4 | 1.8 | 1.2 | 1.3 | **3.6** | 55.2 |

**Table S5. Average ratings across emotion scales as a function of emotion category for nonverbal vocalizations. Values vary between 1 and 7.**

|  | Rating scale | | | | | | | Derived Accuracy |
| --- | --- | --- | --- | --- | --- | --- | --- | --- |
| Group/Emotion | Amusement | Anger | Disgust | Fear | Pleasure | Relief | Sadness |
| Controls |  |  |  |  |  |  |  |  |
| Amusement | **5.2** | 1.3 | 1.4 | 1.6 | 1.7 | 1.8 | 1.6 | 80.3 |
| Anger | 1.2 | **6.1** | 2.7 | 1.5 | 1.5 | 1.4 | 1.1 | 82.5 |
| Disgust | 1.4 | 1.8 | **6** | 1.6 | 1.3 | 1.4 | 1.1 | 87.8 |
| Fear | 1.2 | 1.6 | 1.9 | **5.4** | 1.9 | 1.4 | 1.3 | 77 |
| Pleasure | 1.8 | 1.1 | 1.2 | 1.2 | **5.8** | 2.1 | 1.2 | 88.7 |
| Relief | 1.4 | 1.4 | 1.5 | 1.5 | 2.2 | **4.9** | 1.3 | 68.7 |
| Sadness | 1.2 | 1.2 | 1.4 | 2.4 | 1.7 | 1.6 | **4.9** | 69 |
| Amusics |  |  |  |  |  |  |  |  |
| Amusement | **4.1** | 1.1 | 1.1 | 1.2 | 1.3 | 1.5 | 1.3 | 75.7 |
| Anger | 1.1 | **4.1** | 2.3 | 1.3 | 1.1 | 1.3 | 1.3 | 68.9 |
| Disgust | 1.3 | 1.3 | **3.8** | 1.3 | 1.1 | 1.3 | 1.1 | 71.1 |
| Fear | 1.1 | 1.2 | 1.6 | **3.7** | 1.4 | 1.2 | 1.2 | 66.4 |
| Pleasure | 1.6 | 1 | 1.1 | 1.1 | **3.8** | 1.8 | 1.1 | 71.2 |
| Relief | 1.2 | 1.2 | 1.2 | 1.3 | 1.5 | **3.5** | 1.2 | 71.8 |
| Sadness | 1.1 | 1.2 | 1.3 | 1.8 | 1.1 | 1.3 | **3.5** | 63 |

**Table S6. Average ratings across emotion scales as a function of emotion category for facial expressions. Values vary between 1 and 7.**

|  | Rating scale | | | | | | | Derived Accuracy |
| --- | --- | --- | --- | --- | --- | --- | --- | --- |
| Group/Emotion | Amusement | Anger | Disgust | Fear | Pleasure | Relief | Sadness |
| Controls |  |  |  |  |  |  |  |  |
| Amusement | **6.4** | 1.1 | 1.3 | 1.2 | 1.4 | 1.7 | 1.1 | 97.1 |
| Anger | 1 | **6.5** | 1.7 | 1.7 | 1.1 | 1.2 | 1.2 | 95.6 |
| Disgust | 1.1 | 2 | **4.8** | 2 | 1.1 | 1.3 | 2.3 | 66.8 |
| Fear | 1.1 | 1.8 | 1.4 | **6** | 1.1 | 1.1 | 1.4 | 94.2 |
| Pleasure | 3.5 | 1.1 | 1.2 | 1.1 | **3.4** | 2.7 | 1.2 | 39.8 |
| Relief | 1.4 | 1.2 | 1.2 | 1.1 | 2.8 | **5.2** | 1.5 | 71.6 |
| Sadness | 1.1 | 1.4 | 1.5 | 2.1 | 1.2 | 1.3 | **4.8** | 71.5 |
| Amusics |  |  |  |  |  |  |  |  |
| Amusement | **5.7** | 1.1 | 1 | 1.1 | 1.5 | 1.4 | 1.1 | 88.2 |
| Anger | 1 | **5.6** | 1.6 | 1.4 | 1 | 1 | 1.2 | 93.5 |
| Disgust | 1.1 | 1.7 | **3.5** | 1.5 | 1 | 1 | 2.6 | 50.9 |
| Fear | 1.1 | 1.6 | 1.3 | **4.5** | 1 | 1.1 | 1.3 | 75.6 |
| Pleasure | 3.1 | 1 | 1 | 1 | **2.5** | 1.9 | 1.1 | 31.4 |
| Relief | 1.5 | 1.2 | 1.3 | 1.1 | 1.7 | **3.9** | 1.6 | 58.9 |
| Sadness | 1 | 1.2 | 1.4 | 1.7 | 1 | 1.1 | **4.2** | 71.6 |

Table S7. Average ratings of authenticity and contagiousness for posed laughter, spontaneous laughter, and distractor vocalizations. Values vary between 1 and 7.

| Task/Group | Posed Laughter | Spontaneous Laughter | Distractors |
| --- | --- | --- | --- |
| **Authenticity** |  |  |  |
| Controls | 3.2 | 5.2 | 3.4 |
| Amusics | 3.4 | 4.5 | 3.2 |
| **Contagiousness** |  |  |  |
| Controls | 3.5 | 4.8 | 3.1 |
| Amusics | 3.0 | 3.8 | 2.6 |


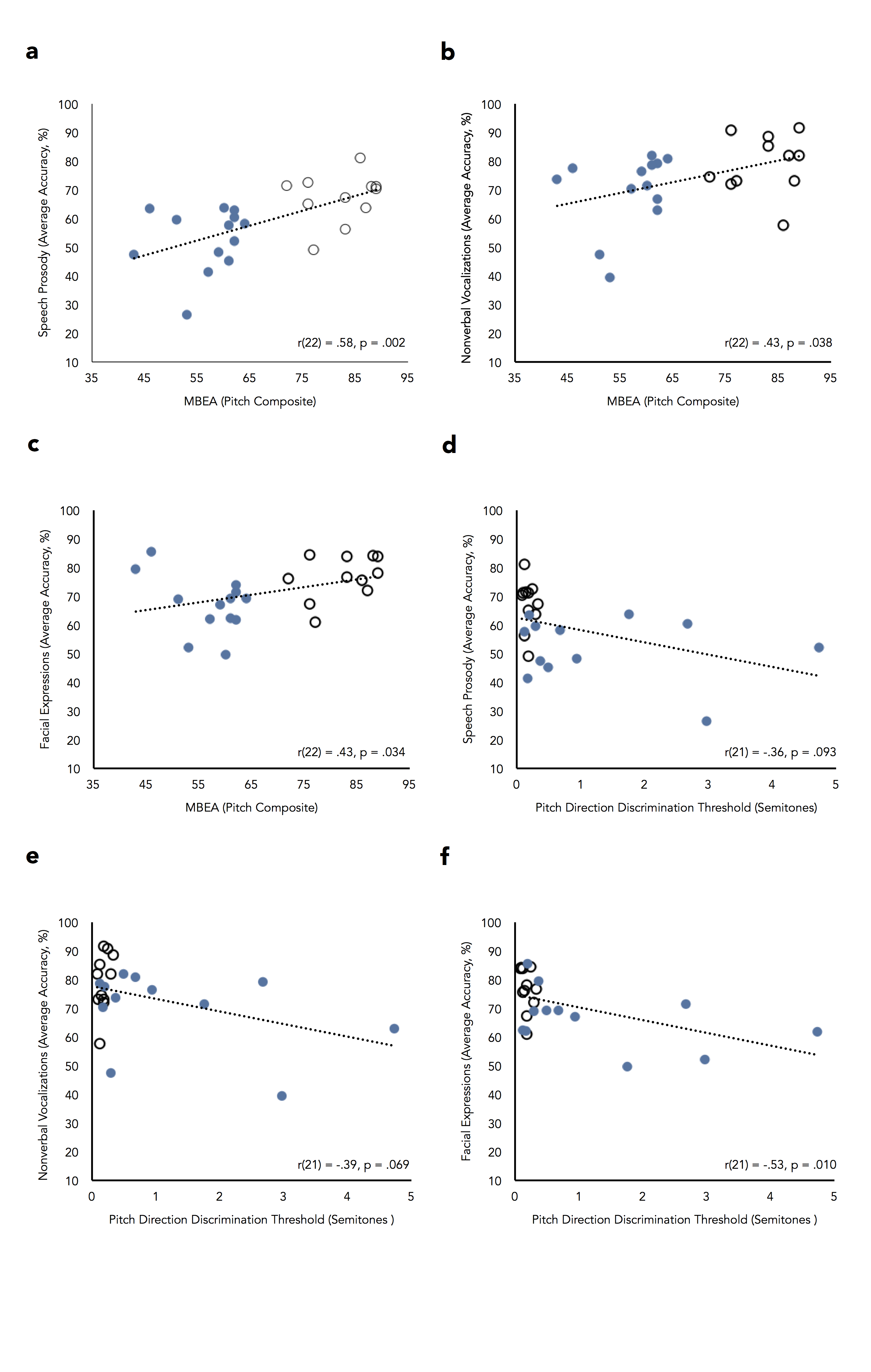
Figure S1. Associations between emotion recognition accuracy (separately for speech prosody, vocalizations, and facial expressions), and performance on the pitch subtests of the Montreal Battery of Evaluation of Amusia (a-c) and on the pitch direction detection task (d-f). Blue circles denote amusics, and white circles controls.

Figure S2. Associations between authenticity and contagiousness evaluations of laughter (as expressed in terms of the effect size of the difference between spontaneous and voluntary laughs), and performance on the pitch subtests of the Montreal Battery of Evaluation of Amusia (a,b) and on the pitch direction detection task (c,d). Blue circles denote amusics, and white circles controls.


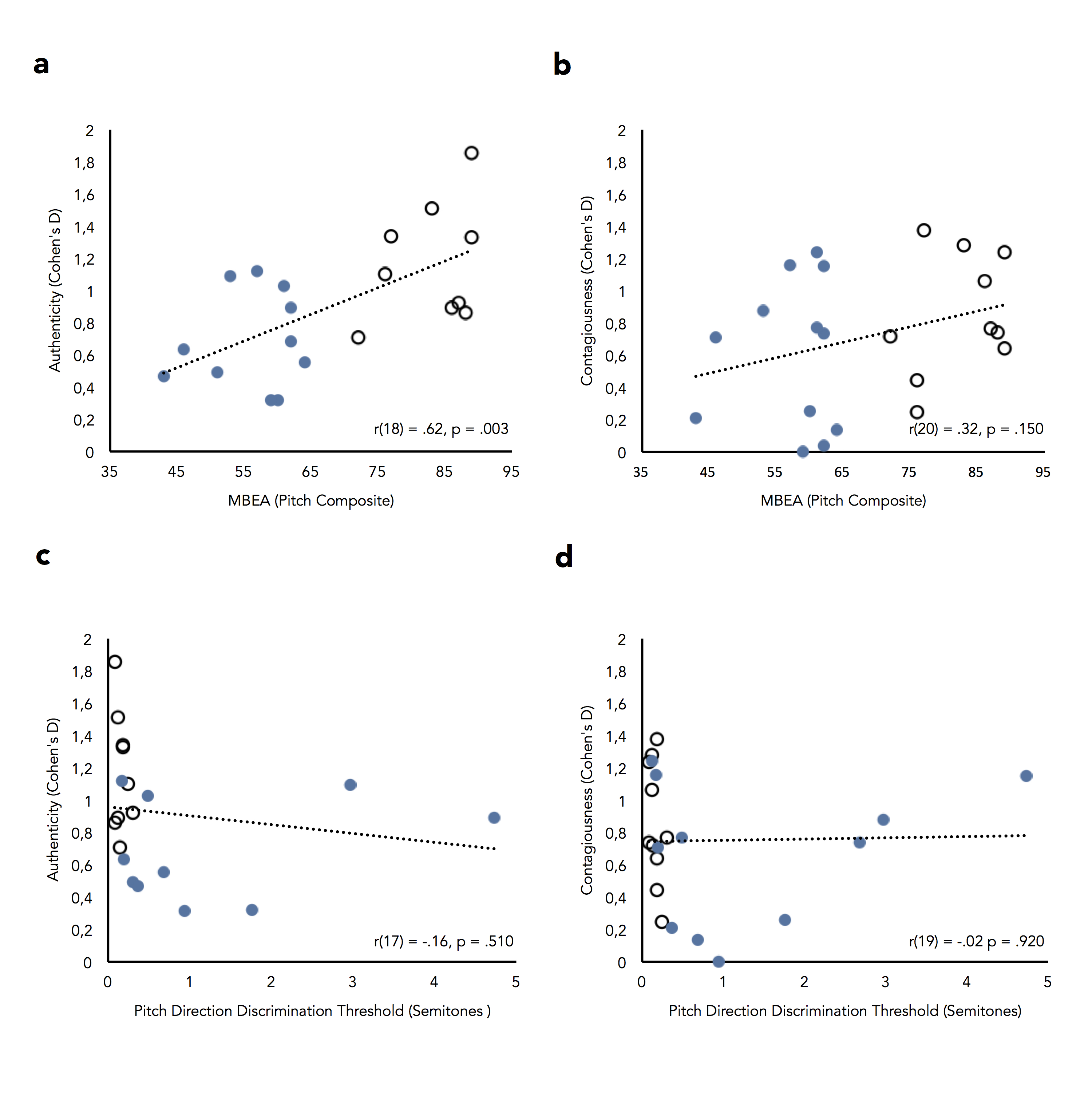


*Recording and validation of emotional speech prosody stimuli*

To create the emotional speech prosody corpus, four drama students were recruited from the Department of Theatre and Performance at Goldsmiths, who were native speakers of British English (two females; aged between 18 and 21 years). They were asked to produce a set of 25 semantically neutral sentences with the intention of communicating emotions via “tone of voice” cues. The sentences were syntactically simple, composed of high-frequency words, and ranged in length between 4 and 7 syllables (*M =* 5.48; *SD* = 1.05; e.g., “Football is a sport”; “She worked in an office”). Individual recording sessions were conducted for each speaker in a sound attenuated booth, and at least two exemplars of each sentence were obtained for each emotion category. The recording procedure was similar to the one used in previous studies^1,2^: after an initial briefing, the speakers were provided with the list of emotions and sentences they had to produce, as well as with a list of illustrative real-life scenarios typically associated with the experience of each emotion (in addition to the aforementioned seven emotion categories, they also produced tenderness and neutrality, not used here). No guidance was provided as to the specific kind of intonation patterns they should make to communicate each emotion, and they were instructed to sound as natural as possible. Individual files were prepared for each sentence and the 20 most recognizable exemplars of each emotion were selected on the basis of 4 judges. A perceptual validation study was conducted to ensure that the stimuli communicated the intended emotions with accuracy rates above chance-level (forced-choice emotion recognition task; *N =* 20; none of these participants took part in the main study with amusic individuals).

Supplementary References:

1. Lima, C. F., Castro, S. L., & Scott, S. K. When voices get emotional: a corpus of nonverbal vocalizations for research on emotion processing. *Behav Res Methods* **45,** 1234-1245 (2013).
2. Castro, S. L., & Lima C. F. Recognizing emotions in spoken language: a validated set of Portuguese sentences and pseudosentences for research on emotional prosody. *Behav Res Methods* **42,** 74-81 (2010).
